# Pregnancy as a Risk Factor of Severe COVID-19

**DOI:** 10.3390/jcm10225458

**Published:** 2021-11-22

**Authors:** Aleksander Celewicz, Marta Celewicz, Michał Michalczyk, Paula Woźniakowska-Gondek, Kamila Krejczy, Marcin Misiek, Rafał Rzepka

**Affiliations:** 1 Department of Gynecology and Obstetrics, Collegium Medicum, University of Zielona Góra, 65-001 Zielona Góra, Poland; mk.celewicz@gmail.com (M.C.); michalmichalczyk123@gmail.com (M.M.); paula.wozniakowska@gmail.com (P.W.-G.); kamila.rasinska@gmail.com (K.K.); rafalrz123@gmail.com (R.R.); 2 Holy Cross Cancer Center, Clinical Gynecology, 25-743 Kielce, Poland; mmisiek@me.com

**Keywords:** SAR-CoV-2, COVID-19, pregnancy, vaccine

## Abstract

Since first being identified in December 2019, severe acute respiratory syndrome coronavirus 2 (SARS-CoV-2) as an etiological agent behind Coronavirus disease 19 (COVID-19), has caused three waves of a global pandemic, with a fourth in progress. Despite its high percentage of asymptomatic and low-symptomatic courses of illness, the SARS-CoV-2 pandemic has claimed a higher death toll than the SARS-CoV and MERS-CoV epidemics because of its high infectivity when compared to the other coronaviruses. High COVID-19 mortality is associated with age and other coexisting morbidities, as well as healthcare quality. According to several studies, pregnant women are at a higher risk of severe COVID-19 infection and adverse pregnancy outcomes (caesarean delivery, pre-term birth, low birth weight, preeclampsia, ICU admission, and need for mechanical ventilation). In our review of recent literature, we focused on the effects of COVID-19 in pregnant women, emphasizing the subcellular pathophysiology of SARS-CoV-2. In this paper, we concentrate on the pathophysiology of sub-cellular changes in COVID-19 and endeavor to highlight the aspects that manifest in physiological pregnancy and potentially create a higher risk of SARS-CoV-2 infection and acute COVID-19 symptoms. Understanding how pregnancy-associated changes can cause a synergistic effect with COVID-19 may point us in the right direction for future prophylaxis and treatment for women undergoing COVID-19 during pregnancy.

## 1. Introduction

Since the first identification of severe acute respiratory syndrome coronavirus 2 (SARS-CoV 2) as an etiological agent behind Coronavirus disease 19 (COVID-19), over 174,574,445 [1] people throughout the world have caught the infection, causing over 3 756,744 deaths worldwide [1]. The WHO declared COVID-19 a pandemic on 11 March 2020. SARS-CoV-2 is a single-stranded RNA virus originating from the same family of viruses that may cause acute respiratory distress syndrome (ARDS), such as the severe acute respiratory syndrome coronavirus (SARS-CoV) and the Middle East respiratory syndrome coronavirus (MERS-CoV) [2]. This group of viruses is characterized by an affinity for the angiotensin II converting enzyme (ACE2) receptor and can replicate in the respiratory tract epithelial cells [3,4]. Despite its high percentage of asymptomatic and low-symptomatic courses of illness, the SARS-CoV-2 pandemic has claimed a higher death toll than the SARS-CoV and MERS-CoV epidemics because of its high rate of infectivity when compared to the other coronaviruses [5]. Identifying asymptomatic carriers proved difficult in the early months of the outbreak, and the virus spread to every country in the world. Thanks to the joint effort of the scientific community and pharmaceutical companies, numerous vaccines against SARS-CoV-2 became available, but despite their effectiveness, the third wave of the pandemic swept across many countries, with the European continent hit severely. The high COVID-19 mortality rate is associated with age and other coexisting morbidities as well as healthcare quality. People with a history of hypertension, cardiac disease, diabetes, COPD (chronic obturator pulmonary disease) and cancer face a higher risk of being infected by a harsher strain of COVID-19 and suffering death [4,6,7].

## 2. Coronaviridae and SARS-CoV-2 Lifecycles

With the 2002–2004 severe acute respiratory syndrome (SARS) epidemic in mind, followed by the MERS epidemic of 2012, we introduce the subject of the morbidity rate in pregnant women infected with these viruses. The SARS epidemic showed that 25–50% of infected pregnant women required intensive care, with the mortality rate estimated at 18–25% [8]. During the latter, 41% of pregnant women were admitted to ICUs, and the mortality rate was 25% [9]. It is worth noting the resemblance between the SARS-CoV (SARS causative virus) and the MERS-CoV (MERS causative virus) when compared with the SARS-CoV-2 (COVID-19). This genetic similarity reaches 79% for SARS-CoV and 59% for MERS-CoV [9,10], which is enough to cause concern in pregnant patients.

SARS-CoV-2 is an enveloped single-stranded RNA virus [11]. The genome size is about 26 to 30 kilobases and the diameter of the virion 80–120 nm [12,13]. The virus is composed of four structural proteins: spike protein (S), membrane protein (M), nucleocapsid protein (N), and envelope protein (E). Studies on the 3D architecture of the virion showed that a bilayer of lipids surrounds the RNA and N proteins. The N protein acts as a protector of the viral genome. With the S protein emerging outwards, the E and M proteins occur in the lipid layers, and are taken from the host cell before the budding of the new virions [14,15,16]. This phenomenon is attributable to the viruses’ inability to produce lipid particles. However, to aid in viral replication and assembly, they can repurpose infected cells’ lipids [2].

**Figure 1:**
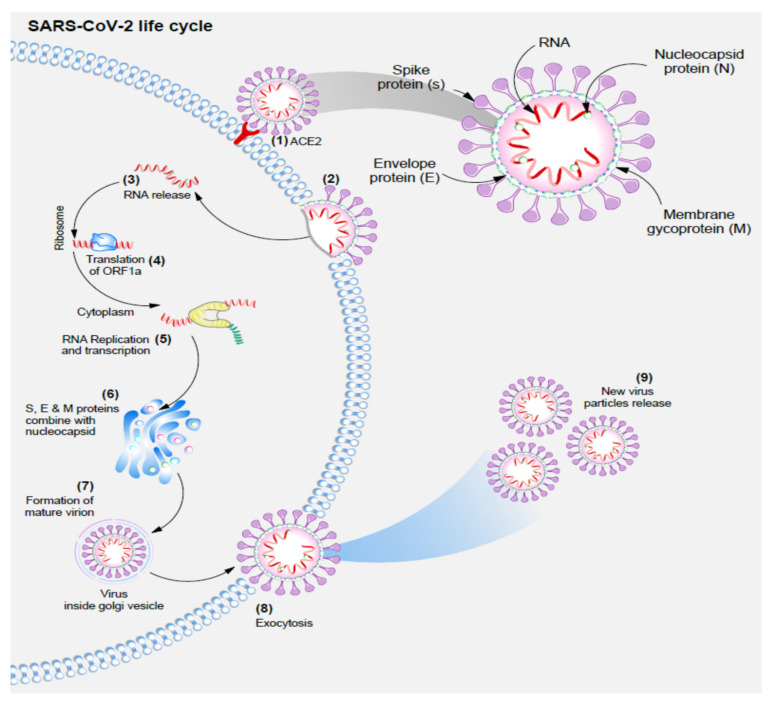
SARS-CoV-2 lifecycle.

We can divide the life cycle of SARS-CoV-2 into several phases, of which the attachment and entry into the cell is the crucial phase [11]. Cell entry into host cells is possible as a result of the strong tropism of SARS-CoV-2 spike protein (S) for the angiotensin II converting enzyme receptor (ACE-2 receptor) [17,18,19,20]. ACE-2 is a cellular transmembrane protein present in most cells, with high expression within type II pneumocytes, enterocytes, endothelial cells, and smooth muscle cells of arteries. Binding to ACE-2 is necessary for host cell proteases to trigger a shift in the conformation of the S proteins (priming) [21,22]. The protease of interest in SARS-CoV-2 infection is the transmembrane serine 2 (TMPRSS2), which carries out the proteolytic cleavage of the S protein and exposes the fusion peptide [17,22]. The fusion peptide can fuse with the targeted cell membrane and pull the viral particle into the cell membrane, thus allowing them to release the viral genome into the host cell [23,24]. Following cell entry, viral RNA is treated as a transcript and facilitates the translation of ORF1a, producing polyprotein pp1a [25]. The structure of ORF1a causes frameshifting within 30% of the ribosomes in the infected cell and produces polyprotein pp1ab [2]. Due to their autoproteolytic function, both proteins generate other non-structural proteins (NSPS) that establish designated behavior in viral replication and form new virions [24,26]. Genomic RNA then becomes a template for the synthesizing of the antisense genome and a new full-length RNA genome of SARS-CoV-2 [12,25]. SARS-CoV-2 undergoes virion assembly in the ER-Golgi intermediate compartment (ERGIC), with the membrane (M) protein of SARS-CoV-2 acting as a mediator in the process [27]. The M protein is crucial for protein binding and forming a sort of virion scaffolding that can attract and attach to other viral protein complexes (M-S (spike) and M-N (nucleocapsid), M-E (envelope) [28,29,30]. Next, the viral particles present in ERGIC are transported by smooth-walled vesicles through the secretory pathway and released via exocytosis [2]. The life cycle of SARS-CoV-2 has been presented on Figure 1.

(1) SARS-CoV-2 enters the cell due to the strong tropism of S protein for ACE-2 receptor;
(2) Fusion of SARS-CoV-2 and the hosts’ cell membrane;
(3) Viral RNA is released into the cell;
(4) Viral RNA is treated as a transcript allowing the translation of ORF1a, which causes frameshifting within 30% of ribosomes, and production of polyprotein pp1ab;
(5) Genomic RNA is then used as a template for the synthesis of an antisense genome, and then a new full-length RNA genome of SARS-CoV-2;
(6) Virion assembly in the ER-Golgi intermediate compartment (ERGIC) with the M protein attracting and attaching to other viral protein complexes M-S, M-N, and M-E;
(7) Mature viral particles are transported by smooth-walled vesicles through the secretory pathway;
(8) Exocytosis of new mature viral particles.

When COVID-19 is severe in its course, the host immune system responds in an uncontrolled process of releasing cytokines (cytokine storm) [31,32]. Cytokines are signaling particles capable of recruiting immune cells as a defense mechanism against infecting agents [32]. In the SARS-CoV-2 infection, this can be upregulated and may drastically increase the leukocyte recruitment to affect the body organs, with the lungs being the most affected, leading to ARDS [33]. The increase in the neutrophil-to-lymphocyte ratio (NLR) can help diagnose clinically high levels of cytokines (or cytokine-storm). Furthermore, SARS-CoV-2 can infect white blood cells (WBC) (lymphocytes, dendritic cells, monocytes, and macrophages), accelerating the cytokine storm [34,35,36].

## 3. Physiological Changes Occurring in Pregnancy That May Contribute to COVID-19 Course

### 3.1. Cardiovascular System

Changes in the cardiovascular system are caused by the placental production of oestrogen and progesterone [37]. The circulating blood volume increases and reaches its maximum between 32 and 34 weeks of pregnancy [38]. The higher increase in serum volume (40–50%) than in red blood cells (20–30%) leads to lower hemoglobin concentrations and hemodilution [39].

Moreover, the heart stroke volume (SV) is increased in pregnant women and reaches its peak at around the 20th week of pregnancy [40,41]. The heart rate (HR) increases by about 10–15 bpm. Venous return is also decreased by up to 24% starting with the II trimester. The increase in blood retention in the venous system and dilation of the arterial capillary vessels may cause renal insufficiency [42]. But the above changes have a protective effect on pulmonary hypertension during the highest increase in circulating blood volume [43,44]. Blood pressure (BP) also decreases during physiological pregnancy as an effect of increasing the activity (and concentration of all components) of the renin–angiotensin–aldosterone system (RAS) [45]. This mechanism does not exclude ACE-2 [46]. Renin is the first hormone to undergo upregulation by the extrarenal release from the decidual tissue and ovaries [47]. In the II trimester, the placenta produces increasing amounts of estrogen, which induces liver synthesis of angiotensinogen and, in effect, increases the levels of angiotensin II (Ang II) [48]. The function of ACE-2 is to hydrolyze angiotensin I (Ang I) to a nonapeptide Ang-(1-9) and Ang II to a heptapeptide Ang-(1-7). Lower BP during pregnancy is achieved by maintaining a balance between increased levels of Ang II and decreased sensitivity to Ang II together with higher levels of Ang-(1-7). Ang-(1-7) has a vasodilatory (RAS modulation), antithrombotic and anti-inflammatory (decreasing TNF-α levels and increasing the anti-inflammatory activity of IL-10) effect [49,50].

### 3.2. Respiratory System

Respiratory system changes occur on every level. The mucosa of the nasopharynx, larynx, trachea, and bronchi is hyperemic and edematous. The causative agent behind it is progesterone, but at the same time, it is responsible for bronchodilation [51]. Progesterone also lowers the carbon dioxide response threshold of the respiratory center [52]. This physiological edema may worsen even during mild infections of the upper respiratory tract [53]. The diaphragm is lifted upwards by the enlarged uterus by up to 4 cm. The dimensions of the chest increase, as does the subcostal angle of the ribs. The result is a decrease in the functional residual capacity (FRC) by 20% to 30%, depending on position [54,55]. A total lung capacity (TLC) is maintained to meet the increased demand for body oxygen (by 20%), and with an unchanged (or only slightly increased) respiratory rate, a higher tidal volume, and a steep increase in minute ventilation with onset in the first trimester [51].

### 3.3. Immune System

Pregnancy demands a unique balance between natural immune tolerance for an allogeneic transplant, the fetus, and preserving an immune response against microbial infections. The exact nature of these is dependent on the gestational age [56]. All of this is necessary to meet the specific developmental fetus at different stages. The changes are not only present locally in the endometrium but are systemic, a factor that can determine the course of COVID-19 to diagnose during a gestational week.

Immunological status during pregnancy induces an increased response against viral infections by exacerbating the activity of natural killer (NK) cells, plasmacytoid dendritic cells (pDC) and monocytes [56,57,58]. Oppositely, Th1 cell activity decreases, and the naïve CD4+ T cells cannot produce Th1 cells at the same level as before pregnancy. Furthermore, the Th2 response is augmented, causing increased morbidity from intracellular pathogens [59]. In the first trimester, the T regulatory lymphocytes (Treg) play a central role in pregnancy implantation and the induction of maternal immunological tolerance for the developing fetus, as they suppress Th1-and Th-17-mediated immune responses [60]. This mechanism generates a further shift in the pregnant woman’s immune system, leading to a Th2-oriented response.

## 4. Manifestation and Outcomes of COVID-19 in Pregnancy

### 4.1. Clinical Manifestations

Clinical manifestations found most commonly in patients with COVID-19 during pregnancy do not differ from the symptoms in non-pregnant patients. Most often, they present with cough (41% of patients), fever (40% of patients), and dyspnea (21% of patients). It is necessary to mention that pregnant patients are likely to have an asymptomatic course of COVID-19 [61,62]. In laboratory parameters, raised C reactive protein (CRP) levels (49% of patients), lymphopenia (33%), and increased leukocyte count (in 26% of patients) were the most common findings [48]. Although we expect young patients to present with mild COVID-19 courses, the pregnant group was more likely to be admitted to an intensive care unit (ICU), with a need for invasive ventilation and extracorporeal membranous oxygenation (ECMO) [61,63] than non-pregnant patients. The risk factors associated with severe COVID-19 and the above complications are a high body-mass index (BMI), chronic hypertension, pre-eclampsia, and pre-existing diabetes [6,61]. All of this points to vascular changes as being the cornerstone of the severe COVID-19 course. As a long-term effect, we may also expect a higher risk of pre-eclampsia or preterm birth (PTB) in pregnant women who have suffered from COVID-19 [61,64].

### 4.2. Vaccination and Pregnancy

After introducing the SARS-CoV-2 vaccines, the elderly and medical personnel were the first to be vaccinated, given they are at the highest risk of infection by a severe COVID-19 course. The latter remain at the highest risk from the virus. With time, the social interest in vaccination has diminished, and in many countries, the younger population shows the lowest vaccination rates. Because of the higher risk COVID-19 poses to pregnant women and of adverse pregnancy outcomes, the American College of Obstetricians and Gynecologists (ACOG), the American Society for Reproductive Medicine (ARSM), the Society for Maternal-Fetal Medicine (SMFM), the Royal College of Obstetricians and Gynecologists (RCOG), and the Centre for Disease Control and Prevention (CDC) have all spoken in favor of vaccination in the pregnant population, non-discriminatory of gestational age [65,66,67,68,69].

Studies reveal encouraging results for the COVID-19 vaccine [69,70]. Millions of vaccinated pregnant women have undergone observation. Therefore, we must determine whether or not to administer the vaccine during the first trimester of the pregnancy or the second and third trimesters. The rationale behind second and third trimester vaccination can be the potential to pass antibodies from the mother to the fetus, thus causing passive immunity in the child after delivery [69,71]. It is now an important determination considering the clinical trials in children under five are ongoing, and the results are pending. The argument against the first trimester vaccination could stem from the fear of the pregnant patient suffering an early gestational miscarriage. In a recent Norwegian study, this correlation was not proven, as the rates of early gestational miscarriage were similar in the vaccinated and unvaccinated populations [72].

Nevertheless, the acceptance of vaccination during pregnancy is dependent on region. In a study performed amidst the third wave of the pandemic (published March 2021), a very high (above 80%) acceptance for vaccination was shown during pregnancy in Mexico and India and below 45% in Russia, the USA, and Australia [73]. This has proven to be more optimistic than reality. The most recent CDC data concerning pregnant population vaccinations (6th November 2021) show that only 13.7% of the pregnant population aged 18–49 was vaccinated before pregnancy, 2.6% received the second dose during pregnancy and 19% were fully vaccinated during pregnancy, adding to a total of 35.3% fully vaccinated pregnant population [74].

## 5. Conclusions and Further Study Directions

Our conclusions from this review are as follows:

Despite the usually mild or asymptomatic course of COVID-19 in younger patients, the pregnant population of the same age has a higher risk of severe and complicated COVID-19. Together with an ever-increasing number of pregnant women with gestational diabetes and obesity, this points to the fact that pregnant women are of critical interest to medical staff and need to be diagnosed and monitored with great care by medical providers.

PTB may not be iatrogenic due to maternal complications in the third trimester (associated with ARDS in the mother).

The vaccination rates among pregnant women are lower than expected in a high-risk population, and broad actions should be taken to promote knowledge of the benefits and safety of COVID-19 vaccines.
